# The Editor's Letter-Box

**Published:** 1918-12-28

**Authors:** 


*To the Editor of* The Hospital.

Sir,—In a recent issue of your paper someone made the statement that to encourage the nurse to articulate was to sow a spirit of disloyalty and discontent. No one on a mere pittance, with very little freedom and entirely minus comforts of any kind, can sustain a spirit of cheerfulness and loyalty over any considerable period. To take an intellectual and enthusiastic interest in one's work one must have speech, social intercourse, and freedom from personal worry. Now, let every reader put these questions to himself and herself. Can one be perfectly healthy with a bare two hours a day off duty? In that time, remember, if no outdoor uniform is allowed, one must get changed from uniform into mufti, and from mufti into uniform again, in order to enjoy a few minutes in the open air. What social intercourse can one enjoy on the luxurious salary of £12 a year, out of which one must buy study-books, foot-wear, pay for the making-up of uniform, and the thousand and one things necessary to make one "respectable"? Can one be free from personal worry with the knowledge that there is absolutely no proviso for old age or sickness, and only the vision of Poor-Law aid and the workhouse looming in the distance? Give any man on the hospital committees £12 a year—sometimes less—let him work from 6.30 a.m. till 2 a.m. the following morning, with only two hours off duty. In the said two hours he must study, get some recreation, and, if very solicitous regarding his health, get some fresh air. Give him also food which is scanty and seldom of the best, and let us see how contented and loyal he will be. How contented! How happy! And how quickly the patients will get well with such a spirit of cheerfulness in their midst! Someone may argue, that the hours on duty are exaggerated. Such is not the case. I have done it, not once, but many times.

I am not a member of the College of Nursing, but I am a trained nurse, and imbued, I suppose, with a spirit of caution. I waited to see what material benefit would accrue to those who so cheerfully gave up their hardly earned guinea. So far I know of no one single instance where the nurse has benefited. They are still being exploited, and the College is still "sitting on the fence." Let the College guarantee to secure for us shorter hours, better pay, protection for our uniform, reasonable travelling expenses, so that we need not hoard every penny of our earnings in order to enjoy our well-earned fortnight's leave, security against the exigencies of old age, sickness, and infirmity, and we will answer its call as readily as we answered the call of our country in its need.—I am, yours truly,

Amor Vitæ.

(Continued on page 276).

